# *ES5* is involved in the regulation of phosphatidylserine synthesis and impacts on early senescence in rice (*Oryza sativa* L.)

**DOI:** 10.1007/s11103-019-00961-4

**Published:** 2020-01-09

**Authors:** Mohammad Hasanuzzaman Rani, Qunen Liu, Ning Yu, Yingxin Zhang, Beifang Wang, Yongrun Cao, Yue Zhang, Md Anowerul Islam, Workie Anley Zegeye, Liyong Cao, Shihua Cheng

**Affiliations:** 1 State Key Laboratory of Rice Biology, China National Rice Research Institute, Hangzhou, 310006 Zhejiang China; 2 China National Center for Rice Improvement, China National Rice Research Institute, Hangzhou, 310006 Zhejiang China; 3 Bangladesh Institute of Nuclear Agriculture, Mymensingh, 2202 Bangladesh; 4 Department of Plant Sciences, University of Gondar, Gondar, Ethiopia

**Keywords:** Phosphatidylserine synthase, Phosphatidylserine, Early leaf senescence 5, Sags, Rice (*Oryza sativa* L.)

## Abstract

Leaf senescence, which affects plant growth and yield in rice, is an ideal target for crop improvement and remarkable advances have been made to identify the mechanism underlying this process. We have characterized an early senile mutant *es5* (early leaf senescence 5) in rice exhibiting leaf yellowing phenotype after the 4-leaf stage. This phenotype was confirmed by the higher accumulation of reactive oxygen species (ROS) and malondialdehyde (MDA), the disintegration of chloroplasts, reduction in chlorophyll content and photosynthetic rate and up-regulation of senescence-associated genes (SAGs) like *Osh36*, *OsI57*, and *OsI85*. Positional cloning revealed that the *es5* phenotype is the result of one base substitution in *ES5*, encoding phosphatidylserine synthase (PSS) family protein, which is involved in the base-exchange type reaction to synthesize the minor membrane phospholipid phosphatidylserine. Functional complementation of *ES5* in the *es5* plants completely restored the wild-type phenotype. Ultra-high-performance liquid chromatography (UHPLC) analysis showed that *es5* plants had increased levels of phosphatidylserine (PS) and decreased level of phosphatidylcholine (PC). These results provide evidence about the role of PS in rice leaf senescence.

**Electronic supplementary material:**

The online version of this article (10.1007/s11103-019-00961-4) contains supplementary material, which is available to authorized users.

## Key message

The *ES5* locus was cloned by map-based cloning that encodes a phosphatidylserine synthase. Mutation in *ES5* caused accumulation of phosphatidylserine which influences premature leaf senescence in rice (*Oryza sativa* L.).

**Electronic supplementary material:**

The online version of this article (10.1007/s11103-019-00961-4) contains supplementary material, which is available to authorized users.

## Introduction

Leaf senescence is an aging process marking the final stage of a plant’s lifecycle. It happens in an age-dependent manner under ideal environments and can be induced early by endogenous or exogenous stimuli, a process termed premature senescence (Schippers 2015; Buchanan-Wollaston et al. 2003). During senescence, the chloroplast is the first target to be dismantled to produce non-fluorescent chlorophyll catabolites (Dodge 1970). As a consequence of chlorophyll pigments disruption, leaf color changes gradually from green to yellow or brown. Macromolecules like nucleic acids, proteins or lipids are degraded, released, recycled and relocated to other organs, especially in storage organs such as the seed (Koyama 2018; Liang et al. 2014). Considering its physiological and agronomic value, senescence could be a potential tool for yield and productivity enhancement in crops through maintenance of proper senescence timing (Thomas and Howarth 2000). Contrarily, premature senility can create a severe loss in agronomic performance ultimately decreasing the crop’s yield (Gan 2014).

Senescence is a developmental phase that is absolutely dependent on the expression of specific genes (Thomas 2013). The genes controlling the senescence process are called senescence associated genes (SAGs), and many advancements have been made to identify the key regulatory genes of senescence in rice. For example, core 2/I branching beta-1,6-*N*-acetylglucosaminyl transferase family gene (Ke et al. 2019), plant spastin gene (Song et al. 2019), ferredoxin-dependent glutamate synthase (Fd-GOGAT) gene (Zeng et al. 2017), pectate lyase precursor (Leng et al. 2017), putative AAA-type ATPase (Huang et al. 2016), Upf1-like helicase (Gong et al. 2019), calcium-dependent protein kinase gene (Wang et al. 2019) are some of the genes regulating senescence in rice.

Lipids are macromolecules essential to cell function. Lipid metabolism, particularly that of membrane lipids, becomes accelerated during senescence and, as a consequence, significant deterioration occurs in the structural and functional integrity of cellular membranes (Thompson et al. 1998). In senescing leaves, membrane lipids are hydrolyzed and metabolized by the action of lipid degrading enzymes such as phospholipase D, phosphatidic acid phosphatase, lytic acyl hydrolase, and lipoxygenase (Thompson et al. 2000). Membrane degradation affects both plasma and intercellular membranes while increasing membrane permeability, leading to the loss of ionic and metabolite gradients, and as a result the cell becomes dysfunctional (Thompson et al. 1998).

Phospholipids contribute substantially to cellular plasma membranes, being distributed throughout the two leaflets of the membrane bilayer (Leventis and Grinstein 2010). Besides the cellular plasma membrane, the membranes of cellular organelles (viz. endoplasmic reticulum, Golgi apparatus, nucleus, mitochondria, peroxisomes and lysosomes) have distinct phospholipid compositions (Vance and Steenbergen 2005). Phospholipids comprise mainly phosphatidylserine (PS), phosphatidylcholine (PC), phosphatidylethanolamine (PE), phosphatidylglycerols (PG), phosphatidylinositol (PI) and phosphatidic acids (PA) (Li et al. 2014). PS is a minor membrane lipid, located in the inner leaflet of the plasma membrane (Delhaize et al. 2002). It accounts only for 2–10% of the total mammalian phospholipids (Jean E. Vance 2008), while the leaves of many plant species contain less than 2% of total phospholipids (Vance and Steenbergen 2005).

Despite the low abundance of PS in membrane lipids, it plays a very important biological role in plant cells. It maintains plasma membrane asymmetry and migration of PS to the outer surface causes asymmetry disruption and cell death (Fadok et al. 1992; O’Brien et al. 1998). PS species contain long-chain fatty acids (LCFA) and very long-chain fatty acids (VLCFAs). VLCFAs have more than 36 carbons in the acyl chain length (Yin et al. 2013). It is speculated that PS is the only VLCFAs in the plasma membrane containing more than 40 carbons (Li et al. 2014), which is believed to maintain the curved shape of the cell membrane, and changes in its composition can be deleterious for the cell (Millar et al. 2000). Carbon chain length of PS is directly related to the plant life span (Li et al. 2014). The overexpression of a wheat PSS gene, involved in PS biosynthesis, in tobacco and Arabidopsis, led to a higher accumulation of PS and directed the cell towards necrotic lesions (Delhaize et al. 2002) implying that PS is associated with cell death. Besides signaling functions in cell death, PS is related to vesicular trafficking, lipid-protein interactions and membrane lipid metabolisms (Vance 2008). It also plays an important role in maintaining the phospholipid homeostasis of the cell (Yin et al. 2013).

The biosynthesis of PS involves two possible pathways: calcium-dependent base exchange type PSS (BE-PSS) and cytidine diphosphate diacylglycerol-dependent PSS (CD-PSS). The first one is present in mammals where PS is synthesized through calcium-dependent base exchange type reactions with PE or PC as substrates. The polar head group of the PE or PC is catalyzed enzymatically by PSS and replaced by l-serine to produce PS. PSS1 releases the choline from PC and PSS2 releases ethanolamine from PE (Leventis and Grinstein 2010). In prokaryotes and yeast, PS is produced through a different pathway (CD-PSS) where CDP-DG is conjugated with serine. Interestingly both pathways have been reported in plants although the process seems to vary among species and organs. The BE-PSS pathway has been reported in the endoplasmic reticulum of castor bean endosperm (Moore 1975) and, in Arabidopsis (Yamaoka et al. 2011), whereas CD-PSS activity was found in spinach leaves (Marshall and Kates 2009) and wheat (Delhaize et al. 2002). Both types of activity have been detected in leek and carrot (Moreau et al. 2002).

In this study, a premature leaf senescent mutant *es5* (early senescence 5) was isolated and characterized. One base substitution was responsible for the altered function of *ES5* which affected PS biosynthesis. Complementation of *ES5* rescued the WT phenotype in the *es5* mutant, indicating that PS plays a role in the control of senescence in rice. These results provide the basis to elucidate the senescence mechanism mediated by PS in rice and create a platform to detect possible functions of PS in higher plants.

## Materials and methods

### Plant materials

The rice mutant, *es5*, was isolated from an ethyl methane sulfonate-treated Japonica background rice variety Jiahe212. For molecular mapping, a segregating generation was developed by hybridizing the *es5* mutant plant with an Indica background rice variety Zhonghui 8015 (ZH8015) (Wang et al. 2018). For fine mapping, an F_3_ segregating population was used. All plant materials, including the two parents and the F_3_ population, were grown in the experimental field of the China National Rice Research Institute at Hangzhou (HZ, 119°54′ E, 30°04′ N) during the rice growing season.

### Phenotypic evaluation

The leaf yellowing phenotype was observed at the 4-leaf stage in the lower leaves of *es5*. Agronomic data were recorded on plant height, number of effective tillers per plant, seed setting rate, thousand grain weight, grain length and width. Agronomic traits were recorded at maturity stage on five individual plants, and the means from three replications were used for analysis.

### Gene cloning and constructs for rice transformation

For fine mapping of the *ES5* locus, 1437 F_3_ homozygous recessive mutant plants were used. Molecular markers were designed by Primer Premier 5.0 software to fine map the candidate region. The sequences of the markers are given in Supplemental Table S1. Gene prediction and sequence analysis were performed using the rice databases of Gramene (http://www.gramene.org) and the Rice Genome Annotation Project (https://rice.plantbiology.msu.edu/analyses_search_blast.shtml). For complementation constructs, an 8260 bp genomic DNA fragment containing the entire *ES5* region, a 2083 bp upstream sequence and a 1376 bp downstream region was amplified from the Japonica rice variety Jiahe212 genomic DNA using KOD FX (Toyobo) and inserted into the EcoRI site of binary vector pCAMBIA1300. For overexpression constructs, a 1272 bp section of full-length CDS was amplified by PCR from the cDNA library of Japonica rice variety Jiahe212 and was subcloned into the BamHI site of the binary vector pCAMBIA1305-GFP. All the constructs were transformed into the wild-type Japonica rice cv. Jiahe212 or *es5* mutant through *Agrobacterium tumefaciens*-mediated transformation. The primer sequences used in this experiment are listed in Supplemental Table S1.

### Pigment content and photosynthetic rate measurement

Chlorophyll and carotenoid contents were measured in the upper second leaf of the WT and *es5* mutant plant for 4 weeks from the date of flowering. To measure these pigments, tissues (100 mg) from the leaf tip of WT and *es5* mutant plants were excised (1–2 cm length), immersed in 10 ml of 80% acetone and then incubated at 25 °C in the dark for 24 h. The optical density of the extracts was measured by spectrophotometry (Beckman Coulter DU800, USA) at 663 nm (the maximum absorption peak of chlorophyll *a*), 645 nm (the maximum absorption peak of chlorophyll *b*) and 470 nm (the maximum absorption peak of carotenoids). Three biological replicates were used per sample. The contents of chlorophyll (Chl*a* and Chl*b*) and carotenoid (Car) in each leaf sample were calculated according to Arnon (1949).

The photosynthetic rate was determined in the flag leaves of WT and *es5* plants with the portable photosynthesis measurement device LI-6400 (Licor, USA) for 4 weeks from the day of flowering. Data were recorded in a sunny day between 9:00 and 11:00 am. The effective intensity was 1200 mmol protons (m^2^ s), the flow rate was 500 mmol s^−1^ and the relative humidity was 65–80%.

### Histochemical analysis

Evans blue staining was performed to observe cell death as described by Kong and Li (2011). Fresh leaves of WT and *es5* mutant plants were immersed into 0.25% Evans blue solution (0.25 g Evans blue dye was dissolved in 100 ml of 0.1 M CaCl_2_ solution at pH 5.6) and kept in the dark overnight at 25ºC. The next morning the leaves were washed with water and boiled for 10 min in 96% ethanol to remove chlorophylls. Then the leaves were transferred into 60% glycerol and examined and photographed by camera (D800, Nikon, Japan).

Hydrogen peroxide was visually detected in leaves using 3,3′-diaminobenzidine (DAB) staining following the method described by Thordal-Christansen (1997). Leaf samples were soaked in DAB overnight in the dark at 25 °C. Then they were boiled in 95% ethanol for 10 min to remove chlorophylls. Finally, the samples were transferred into absolute ethanol and examined and photographed by camera (D800, Nikon, Japan).

### Transmission electron microscopy

The upper second leaves of the WT and *es5* plants were collected at tillering stage and cut into small pieces. The samples were fixed with 2.5% glutaraldehyde in phosphate buffer (0.1 M, pH 7.0) for more than 4 h at 4 °C. The samples were washed three times in phosphate buffer and post fixed with 1% OsO_4_ in phosphate buffer for 1–2 h. Then the samples were washed 3 times with phosphate buffer and dehydrated by a graded series of ethanol (30 to 100%) for 15–20 min at each step. Finally, the samples were transferred to absolute acetone for 20 min. The samples were placed in a series of resin spur mixtures overnight. Specimens were placed in Eppendorf microtubes containing spur resin and heated at 70 °C for more than 9 h. Specimens were sectioned in Leica EM UC7 ultratome and sections were stained with uranyl acetate and alkaline lead citrate for 5 and 10 min, respectively, and observed by TEM (Hitachi Model H-7650).

### Measurement of enzymatic activity and senescence related parameters

Malondialdehyde (MDA) content, Super Oxide Dismutase (SOD) and Catalase (CAT) activity and Soluble Protein (SP) content of wild type and mutant leaves were measured from the day of flowering up to 21 days at 7 days intervals. The SP content, MDA content, SOD and CAT activity of the leaves were determined using the Coomassie brilliant blue protein determination kit (A045-2), the thiobarbituric acid (TBA) assay kit (A003-1), the SOD assay kit (A001-1) and the CAT assay kit, respectively; all of which were obtained from the Nanjing Jiancheng technology company (China).

### Gene expression analysis

Total RNA was extracted from different parts of wild type and *es5* mutant plants using RNAprep Pure kit for plants (Tiangen, China). RNA was converted to first-strand cDNA by the ReverTra Ace qPCR RT kit (Toyobo, Japan). qRT-PCR was performed using SYBR premix Ex Taq II (Takara, Japan) in the Light Cycler 480 II (Roche, Sweden) according to the manufacturer’s instructions. To analyze the transcript levels of different genes, the 2^−ΔΔct^ method was applied where the values are the means of three biological replicates and *OsActin* was used as internal control. Gene specific primers are given in Supplementary Table S1.

### GUS assay

A 2-kb native promoter of *ES5* was amplified and inserted to the pCAMBIA 1305 vector to create the *ES5*-pro:GUS construct. The tissues from transformed plants were then used for GUS staining according to a previous method with some modifications (Jefferson 1987). In brief, rice tissues were stained with GUS staining solution (50 mM PBS buffer; 10 mM EDTA, pH 8.0; 0.1% Triton X-100; 1 mg/mL X-gluc; 1 mM potassium ferricyanide; 1 mM potassium ferrocyanide). After incubating at 37 °C in the dark for 10 h, the chlorophyll in the tissues was removed by boiling in 95% ethanol.

### Protein sequence alignment and protein domain identification

Homologous protein sequences of *ES5* were obtained from rice, Arabidopsis, maize and tobacco from the NCBI BLAST server (https://www.ncbi.nlm.nih.gov/). Amino acid sequence alignments were conducted following the ClustalW method from Clustal Omega server (https://www.clustal.org/). The transmembrane domains of *ES5* were predicted through TMHMM v2.0 (https://www.cbs.dtu.dk/services/tmhmm-2.0/). The phylogenetic tree was generated from Geneious software (https://www.geneious.com) following the neighbor-joining method. The following accessions were used in phylogenetic analysis: *Corchorus olitorius* (OMO49472.1), *Theobroma cacao* (EOX99944.1), *Cephalotus follicularis* (GAV87842.1), *Vigna unguiculata* (QCE08564.1), *Rosa chinensis* (XP_024193920.1), *Populus alba* (TKR84883.1), *Handroanthus impetiginosus* (PIN01155.1), *Camellia sinensis* (XP_028080563.1), *Musa acuminata subsp. malaccensis* (XP_009393589.1), *Apostasia shenzhenica* (PKA65308.1), *Oryza brachyantha* (XP_006654757.1), *Setaria italica* (XP_004961292.1), *Sorghum bicolor* (XP_002440211.1), *Zea mays* (NP_001149567.1), *Brachypodium distachyon* (XP_003567934.1), *Hordeum vulgare subsp. vulgare* (BAJ95475.1), *Nicotiana tabacum* (AHM22937.1), *Arabidopsis thaliana* (AT1G15110.2).

### Measurement of PSS

After sampling the leaf tissue was weighed and cut into small pieces and put into phosphate-buffered saline (PBS) solution. Then the samples were rapidly frozen in liquid nitrogen and maintained at 2–8 °C after melting. Samples were then centrifuged for 20 min at 2000 rpm. The PSS content was measured using a Plant Phosphatidylserine Synthase (PTDSS) ELISA kit (mlbio) according to the user manual.

### Measurement of phospholipids

For phospholipid extraction, 25 mg of sample was weighed to a microtube, homogenized at 30 Hz for 4 min and sonicated for 5 min in an ice-water bath. The homogenization and sonication cycles were repeated twice. 200 μL water, after 30 s vortex, the samples were homogenized at 30 Hz for 4 min and sonicated for 5 min in ice-water bath. The homogenization and sonication cycle were repeated for 2 times. 480 μL extraction solution (methyl-tert-butyl ether (MTBE): methanol = 5:1) was added and the samples were again sonicated for 10 min in an ice-water bath. Then the samples were incubated at − 40 °C for 1 h and centrifuged at 10,000 rpm for 15 min at 4 °C. 300 μL of the supernatant was transferred to a fresh tube and dried in a vacuum concentrator at 37 °C. Then, the dried samples were reconstituted in 200 μL of 50% methanol in dichloromethane by sonication in ice for 10 min. The solution was then centrifuged at 13,000 rpm for 15 min at 4 °C, and 75 μL of supernatant was transferred to a fresh glass vial for LC/MS analysis. The UHPLC separation was carried out using a 1290 Infinity series UHPLC System (Agilent Technologies), equipped with a Kinetex C18 column (2.1 × 100 mm, 1.7 μm, Phenomen). The Triple time-of-flight (TOF) mass spectrometer was used to acquire MS/MS spectra on an information-dependent basis (IDA) during LC/MS analysis. An in-house program, LipidAnalyzer, was developed using R for automatic data analysis. The raw data files were converted to mzXML format using the ‘msconvert’ program from ProteoWizard (version 3.0.6150). Then, the mzxML files were loaded into LipidAnalyzer for data processing. Peak detection was carried using CentWave algorithm with the MS/MS spectrum. Lipid identification was achieved through a spectral match using an in-house MS/MS spectral library.

### Statistical analysis

All the results are expressed as mean values ± SD based on three biological replicates. Statistical significance was assessed using Student’s unpaired *t* test (*P* < 0.05). Single asterisk (*) and double asterisk (**) represents a significance level of 5 and 1%, respectively. The statistical analysis of gene relative expression levels, physiological traits and agronomic traits in this study all follow the methods described above.

## Results

### Phenotypic characterization of *es5*

**Fig. 1:**
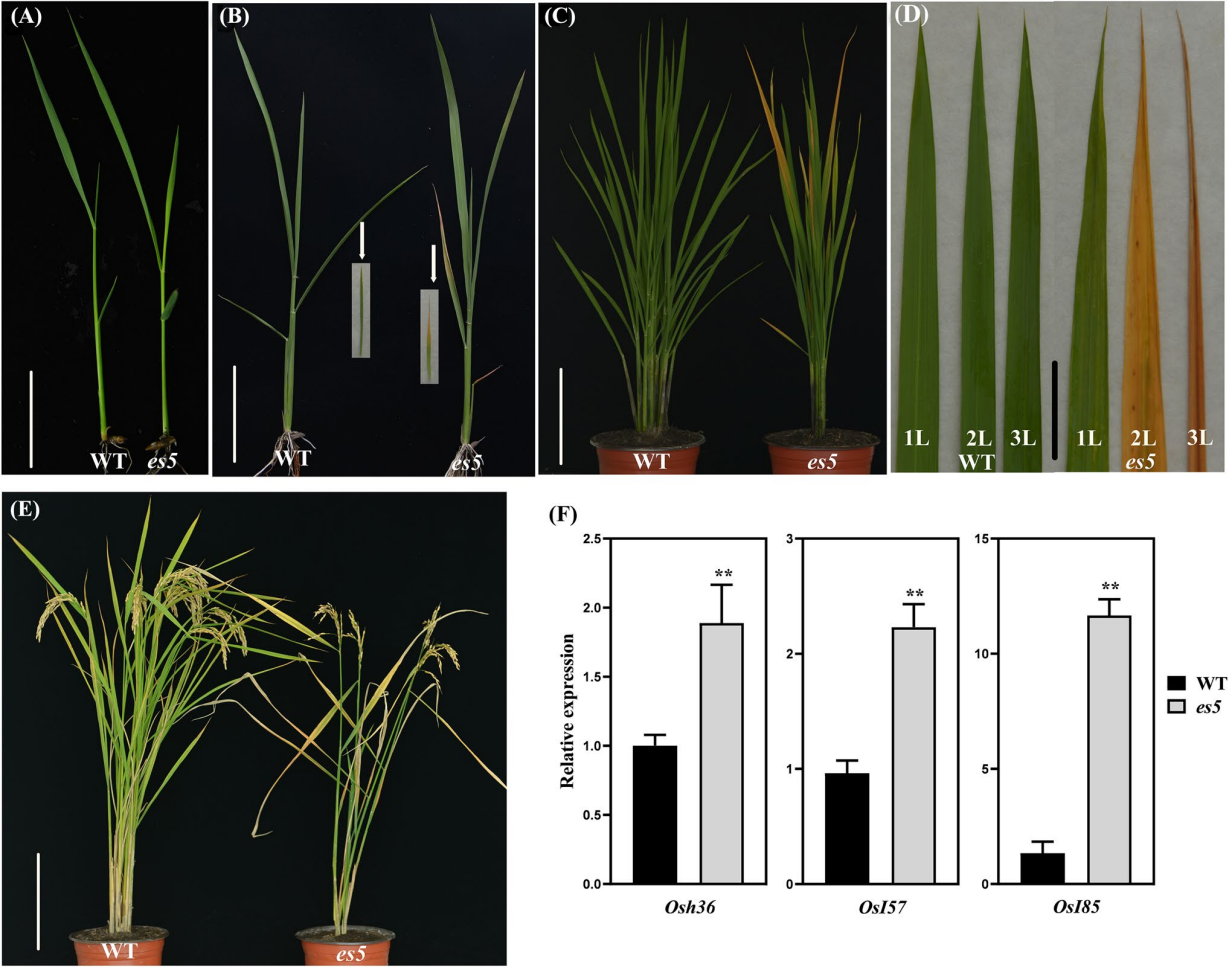
Phenotypic characterization of *es5* plants. **a**–**e** Phenotypes of the wild-type and *es5* mutant seedlings at 3-leaf stage (**a**), seedlings at 21 days after sowing (**b**), plants at tillering stage (**c**), upper three leaves at tillering stage (**d**) and plants at maturity stage (**e**); scale bar = 3, 5, 30, 4 and 40 cm, respectively at the figures of **a**–**e**. **f** Relative expression of the senescence associated genes. Values are mean ± SD of three biological replicates; p ≤ 0.01; Student’s *t* test

There was no visible phenotypic discrepancy between wild-type and *es5* mutant plants before 4-leaf stage (Fig. 1a). The leaf yellowing phenotype of the *es5* mutant commenced from the lower leaf after 4-leaf stage (Fig. 1b) and the upper functional leaves exhibited gradual yellowing phenotype from tillering stage (Fig. 1c, d). After heading, the leaf yellowing rate was very fast and became severe at maturity stage which resulted in 5–7 days early maturation in *es5* plants compared to wild-type (Fig. 1e). This early senescence also affected agronomic traits negatively (Fig. S1). The *es5* mutant became shorter and produced 4.83 ± 0.65 effective tillers plant^−1^, which is almost 44% lower than the wild-type (Fig. S1a, b). Seed setting rate, thousand grain weight and grain length were also significantly reduced in the *es5* mutant plants (Fig. S1c–e). Seed setting rate was 58.8 ± 1.75% in the *es5* plants which is much lower than the wild-type (89.5 ± 0.7%). Thousand grain weight was 27.3 ± 0.2 g, which is lighter than the wild-type (29.8 ± 0.17 g). Grain length also reduced in the mutant plants up to 5%. The mRNA levels of yield related genes *GS3* and *GS5* had also altered expression levels in the *es5* mutant plants (Fig. S2). In accordance to phenotypic observations, mRNA levels of senescence associated genes (*Osh36*, *Osl57* and *Osl85*) were up-regulated in the *es5* plants (Fig. 1f).

### Physiological characterization

**Fig. 2:**
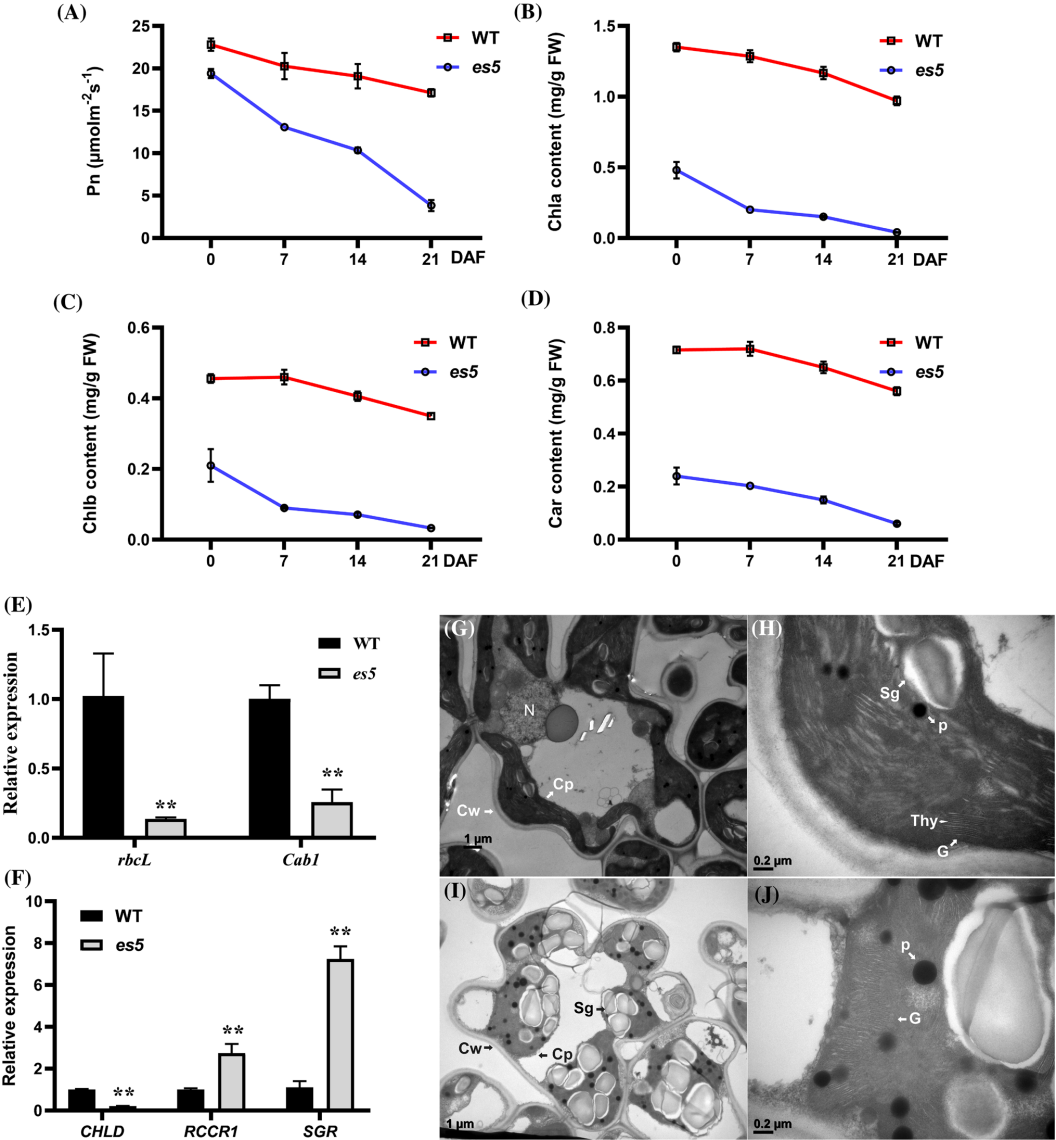
Physiological characterization of *es5* plants. **a**–**d** kinetic analysis of photosynthetic rate, Chl*a*, Chl*b* and carotenoids. Photosynthetic rate was measured in the flag leaves and chlorophyll pigments in the upper second leaves. **e**–**f** Relative expression of photosynthesis related genes (*rbcL* and *Cab1*) and chlorophyll biosynthesis and degradation related genes (*CHLD*, *RCCR1* and *SGR*). **g**–**j** TEM of wild-type (**g**, **h**) and *es5* mutant (**i**, **j**) leaves at tillering stage. *Cp* chloroplast, *Cw* cell wall, *P* plastoglobuli, *G* granum, *Thy* thylakoid, *Sg* sugar granule and *N* nucleus. Values are mean ± SD of three biological replicates; p ≤ 0.01; Student’s *t* test

Photosynthetic rate and chlorophyll content are the two major characteristic physiological indicators to evaluate the degree of senescence in plants. To assess the physiological basis of early senescence in *es5* plants, these two physiological indicators were measured in the flag or upper second leaves from the day of flowering to up to 21 days after flowering. It was observed that the photosynthetic rate was lower in the *es5* plants compared to wild-type and decreased gradually towards maturity (Fig. 2a). Photosynthesis related genes viz*., rbcL* and *Cab1* were also down-regulated in the *es5* plants indicating lower photosynthetic activity in the mutant (Fig. 2e). Chlorophyll contents were also decreased in the same manner but the content of Chl*a*, Chl*b* and carotenoid were very low compared to wild-type. On the day of flowering Chl*a*, Chl*b* and carotenoid contents were only 35, 46 and 33% of wild-type, respectively in the upper second leaves of *es5* plants and these were hardly detectable at 21 days of flowering as the declining rate was very high (Fig. 2b–d). The Chl*a*, Chl*b* and carotenoid content were also less at the senescence initiating 4-leaf stage in the *es5* mutant seedlings (Fig. S3c–e). These data are consistent with the down-regulation of chlorophyll biosynthesis gene (*CHLD*) and up-regulation of chlorophyll degradation related genes (*RCCR1* and *SGR*) (Fig. 2f). In addition to these results, electron microscopy revealed the presence of well-developed mesophyll cells in the wild-type leaves compared to *es5*. Intact thylakoid and granum structure with tightly stacked lamellae in the grana were observed in the wild-type leaf cells (Fig. 2g, h). In addition, chloroplasts were more condensed. In contrast, *es5* leaf cells had degenerated thylakoid and granum structure as well as less condensed chloroplast structure (Fig. 2i, j). The mutant leaf cells also had a greater number of osmophilic plastoglobuli, which is generally a result of the release of chloroplast lipids in droplet form. These results indicate an abnormal chloroplast development in mutant leaf cells.

### ROS accumulation and cell death scenario

**Fig. 3:**
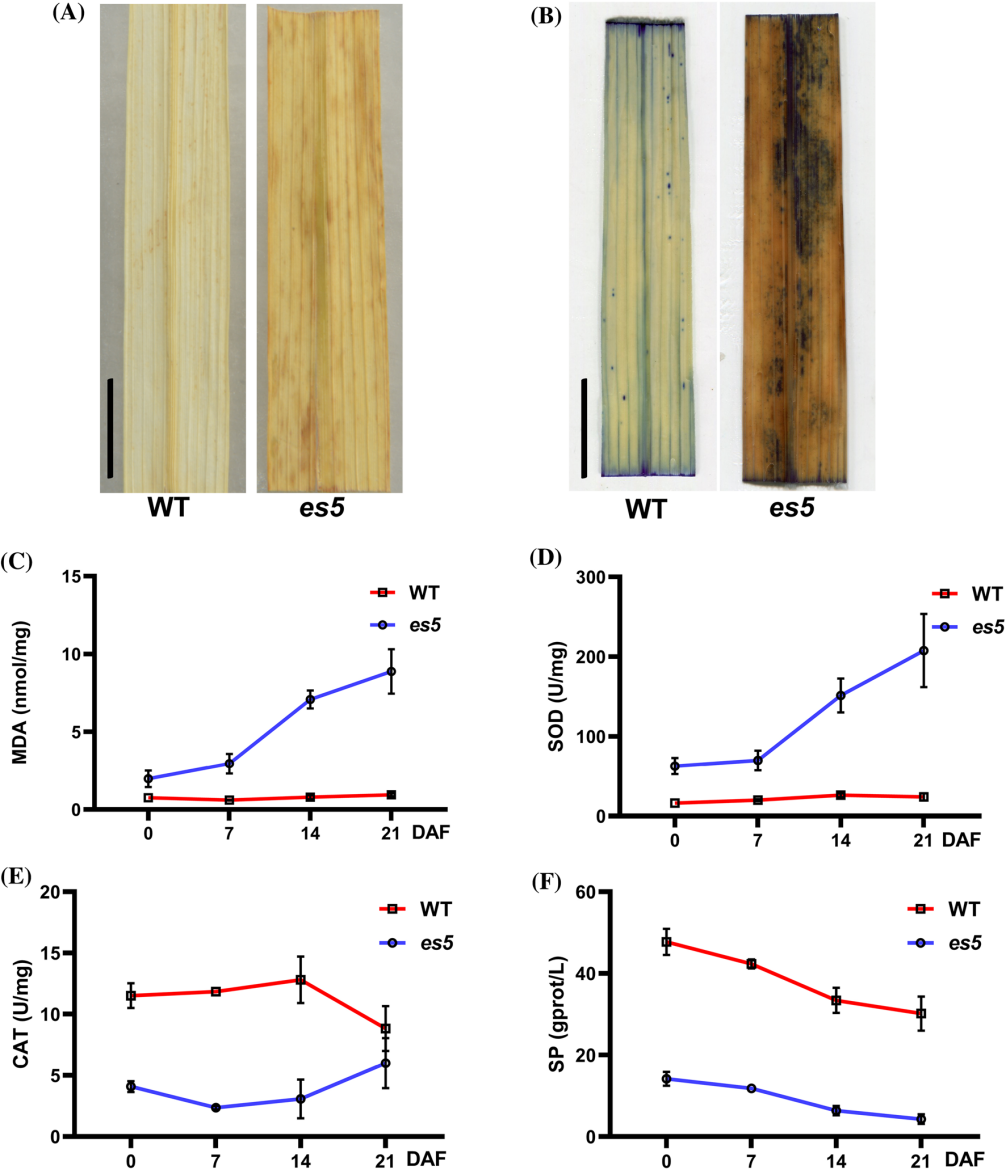
ROS accumulation and its scavenging. **a**–**b** Histochemical detection of H_2_O_2_ accumulation by DAB staining (**a**) and dead cells by Evans blue staining (**b**); scale bars = 2 cm. **c**–**f** Time-course analysis of early senescence indicators- MDA, SOD, CAT and SP contents in the upper second leaves. The data represent the means ± SD of three biological replicates

In senescing leaves H_2_O_2_ accumulation increases due to oxidative reactions (Gechev et al. 2006). In the presence of peroxidases, H_2_O_2_ can react with DAB and produce a reddish-brown polymer precipitate. DAB staining was performed to investigate the H_2_O_2_ accumulation level in the wild-type and mutant leaves. Large amounts of reddish-brown precipitate, indicating the accumulation of H_2_O_2_, was observed in the mutant leaves, but not in the wild-type leaves (Figs. 3a and S3a).

Senescence-associated cell death or membrane damage are considered irreversible processes. To investigate the cell death scenario, wild-type and *es5* mutant leaves were stained with Evans blue staining solution, which can only enter dead cells. After Evans blue staining, dark blue spots were found on the *es5* mutant leaves, suggesting cell death in these samples (Figs. 3b and S3b).

MDA is produced and accumulated in the cell due to membrane lipid peroxidation (Leshem 1988). High accumulation of MDA causes cell membrane damage, leading to cell death. It was observed that MDA accumulation was always higher in the *es5* mutant leaves and increased rapidly after 7 DAF and gradually every week thereafter. This trend was not observed in wild-type leaves (Fig. 3c). MDA content was also higher in the *es5* mutant at the senescence initiating 4-leaf stage (Fig. S4f). This result implies that there is significant lipid peroxidation happening in the mutant samples, which provides further evidence that *es5* mutants confronted oxidative stress and accumulated ROS in response.

During oxidative stress two major antioxidative enzymes SOD and CAT, are increased to scavenge the produced ROS, enabling plants to battle against aging. If the antioxidative enzyme activity is lower than ROS production, plants face aging (Grzegorz 1997). In our experiments, SOD was always higher in *es5* mutant plants and increased gradually after 7 DAF whereas CAT activity was significantly lower compared to wild type (Fig. 3d, e). At the senescence initiating 4-leaf stage, SOD level was higher but CAT level was similar to the wild-type plants (Fig. S4g, h). Increased SOD activity indicates that *es5* plants may responded actively to O^2−^ accumulation by producing more H_2_O_2_. However, CAT activity may not be sufficient to scavenge the additional H_2_O_2_, leading to H_2_O_2_ accumulation in leaves.

Another senescence indicator is the reduction in total SP content. During senescence SP decreases due to the reduction of scavenging activity (Lim and Nam 2005). In this study, SP content in the *es5* mutant was lower than the wild-type plants and decreased gradually (Figs. 3f and S3i), indicating the initiation of nutrient remobilization in the *es5* plants.

### Mapping-based cloning and functional analysis of *ES5*

To develop the mapping population a cross was made between *es5* mutant and ZH8015. The genetic analysis confirmed that the premature leaf yellowing phenotype was controlled by a single homozygous recessive gene and the candidate region of that gene was preliminary mapped on the long arm of chromosome 5 in between two SSR markers RM3486 and RM3664 with a physical distance of 2 Mbp (Wang et al., 2018).

**Fig. 4:**
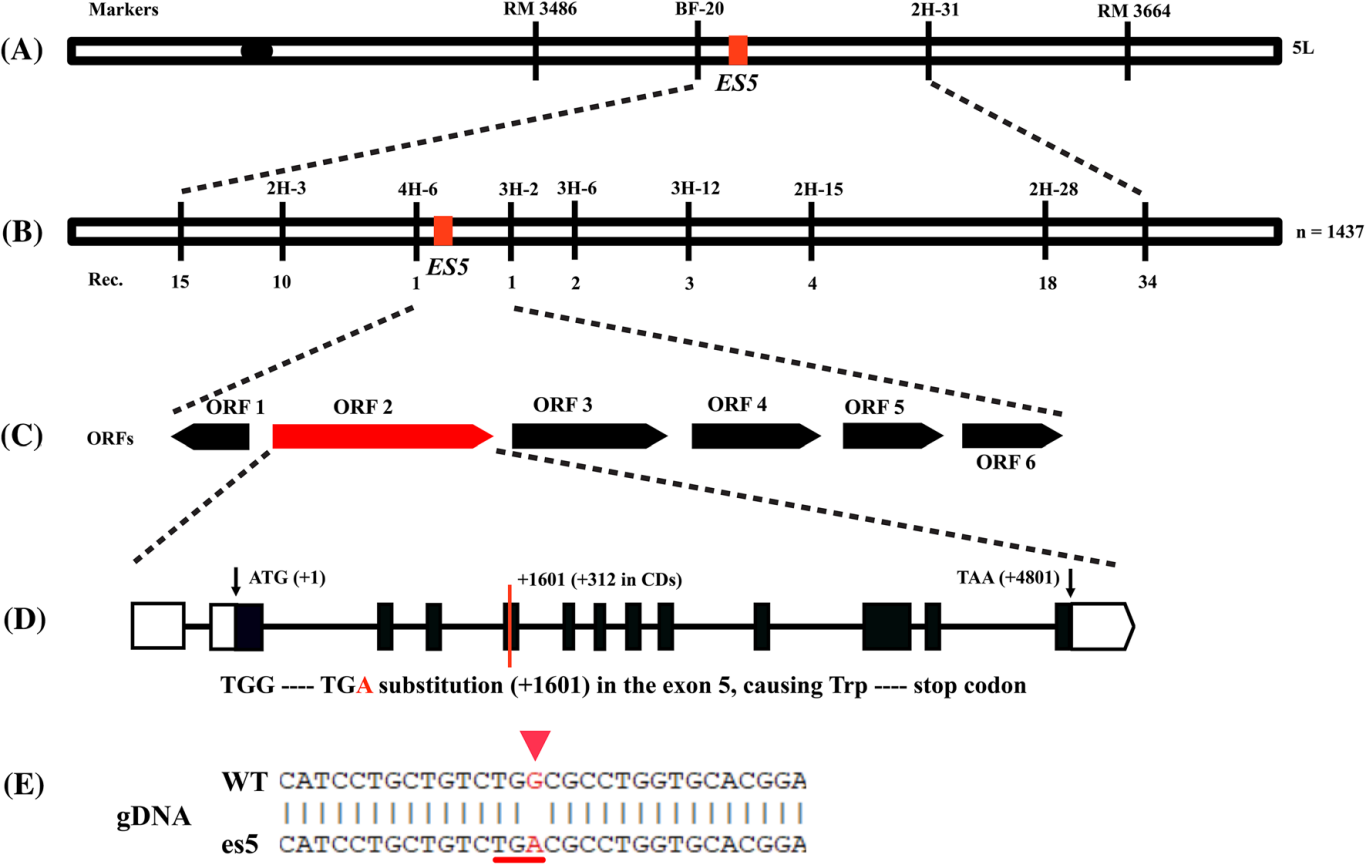
Map-based cloning of *ES5* gene. **a** Preliminary mapping of *ES5* between the SSR markers RM 3486 and RM 3664 on the long arm of chromosome 5. **b***ES5* gene was fine mapped to an interval of 37.57 kb region by 1437 mutant individuals. **c** Six putative ORFs were annotated in the 37.57 kb region, and gDNA sequencing revealed that ORF2 had one base substitution from G-to-A and creation of a premature stop codon (**d**, **e**)

To fine map the region 10 InDel primers were designed and used to survey 1437 homozygous recessive *es5* mutants. Finally, the region was narrowed down to a physical distance of 37.57 kb between the InDel primers 4H-6 and 3H-2 (Fig. 4a, b).

**Fig. 5:**
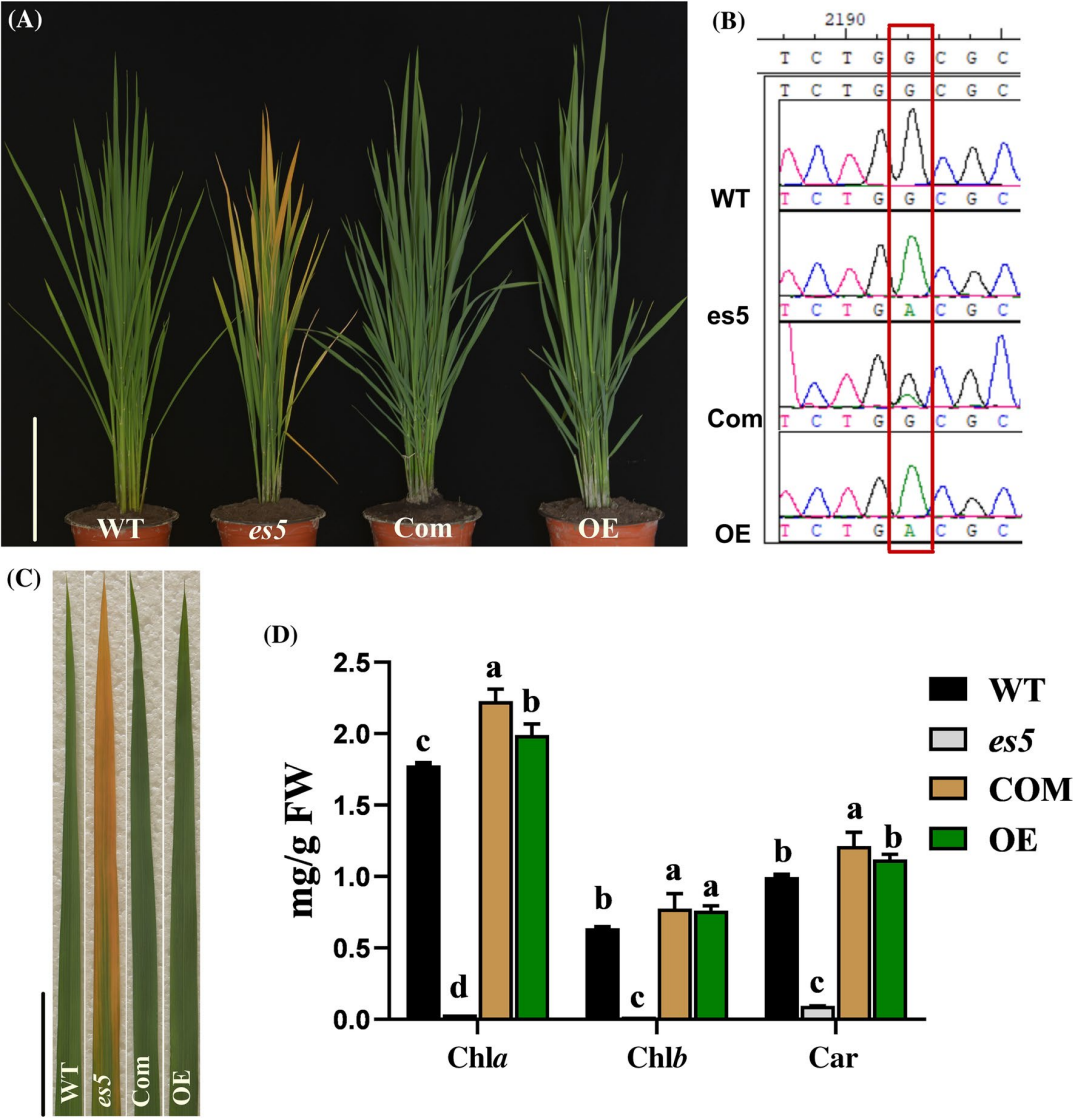
Complementation and overexpression analysis of *ES5* gene in *es5* mutant. a. Phenotype of WT, *es5* mutant, complementation and overexpression plants at tillering stage; scale bar = 30 cm. b. Sequencing analysis of the *ES5* in T_0_ transgenic plants. c. The leaf color from each plant; scale bar = 5 cm. d. Photosynthetic pigment contents of leaf WT, *es5*, complementation and overexpression plants. Data = mean ± SD (n = 3). Letters in the figure indicate the result of multiple comparison test; a, b, c indicate the significant differences on 0.05 level

According to the Rice Genome Annotation Project database (https://rice.plantbiology.msu.edu) the fine mapped region harbors six putative open reading frames (ORFs) (Fig. 4c). All of these ORFs were amplified from the genomic DNA (gDNA) of *es5* mutant and wild-type plants by PCR and sequenced. The DNA sequencing results showed that the *es5* mutant has a ‘G-to-A’ substitution at position 1601 in the ORF2. This result was confirmed by cDNA sequencing. Thus, the locus ORF2 (LOC_Os05g48060), termed from here on as *ES5*, was considered as the targeted gene most likely responsible for the mutant phenotype. The length of *ES5* is 5647 bp with 13 exons and 12 introns. The full-length cDNA is 1980 bp, containing 5′-UTR (455-bp), a coding sequence (1269-bp) and a 3′-UTR (256-bp). The CDS is predicted to encode a polypeptide of 422 amino acids. The G-to-A substitution is present in the 5th exon. This one base substitution created a new stop codon ‘TGA’, which was before a tryptophan coding sequence ‘TGG’ (Fig. 4d, e). To validate the function of *ES5*, the complementary vector pCAMBIA1300-*ES5* was transformed in the *es5* calli through *Agrobacterium tumefaciens*-mediated transformation. A total of twelve transgenic plants were obtained and all of them displayed regular green phenotype as the wild-type Japonica rice cv. Jiahe212 (Fig. 5a, c). Analysis of independent T_0_ transgenic plants by PCR, using primers flanking the mutation site following with sequencing, exhibited a double peak (‘G’ and ‘A’) at the mutation site, corroborating the presence of the normal *ES5* gene sequence (Fig. 5b).

To verify whether any other fragments from the *ES5* gene caused the genetic complementation and recovery of leaf color in the transgenic complemented plants, a fragment containing the full-length CDS of the *ES5* gene was ligated to a ubiquitin promoter for overexpression analysis in *es5*. Overexpression vector pCAMBIA1305-GFP-*ES5* was introduced in the *es5* calli via *A. tumefaciens* transformation. A total of 10 transgenic plants were obtained and they showed the same phenotype as wild-type Japonica rice cv. Jiahe212 (Fig. 5a, c). Meanwhile the complementation and overexpression lines did not show any differences in growth and development compared to the wild-type plants. Additionally, the *es5* mutant had significantly lower level of *Chl*a, *Chl*b and carotenoid contents compared to the wild-type, complementation and overexpression lines (Fig. 5d). The *es5* leaf had more accumulation of H_2_O_2_ and cell death compared to complementation, overexpression and wild-type leaves (Fig. S4a and b). There were more accumulation of MDA and SOD content in the *es5* mutant leaves compared to complementation, overexpression and wild-type leaves (Fig. S4c, d). Moreover, complementation and overexpression plants had more CAT activity than the wild-type and *es5* mutant leaves (Fig. S4e). In addition, SP content of complementation and overexpression lines were similar to wild-type and higher than *es5* mutant plants (Fig. S4f). These results indicate that the candidate *ES5* is responsible for the *es5* phenotype.

The expression levels of *ES5* in the complementation and overexpression lines were approximately two and three-fold higher than the wild-type plants (Fig. S5a). Agronomic traits like plant height, tiller number, grain length and 1000-grain weight were recovered in the complementation and overexpression lines (Fig. S5b–f). Complementation and overexpression lines had higher tiller number whereas, grain length and 1000-grain weight were slightly higher in the overexpression lines compared to the wild-type plants (Fig. S5e, f).

### *ES5* is highly expressed in leaf

**Fig. 6:**
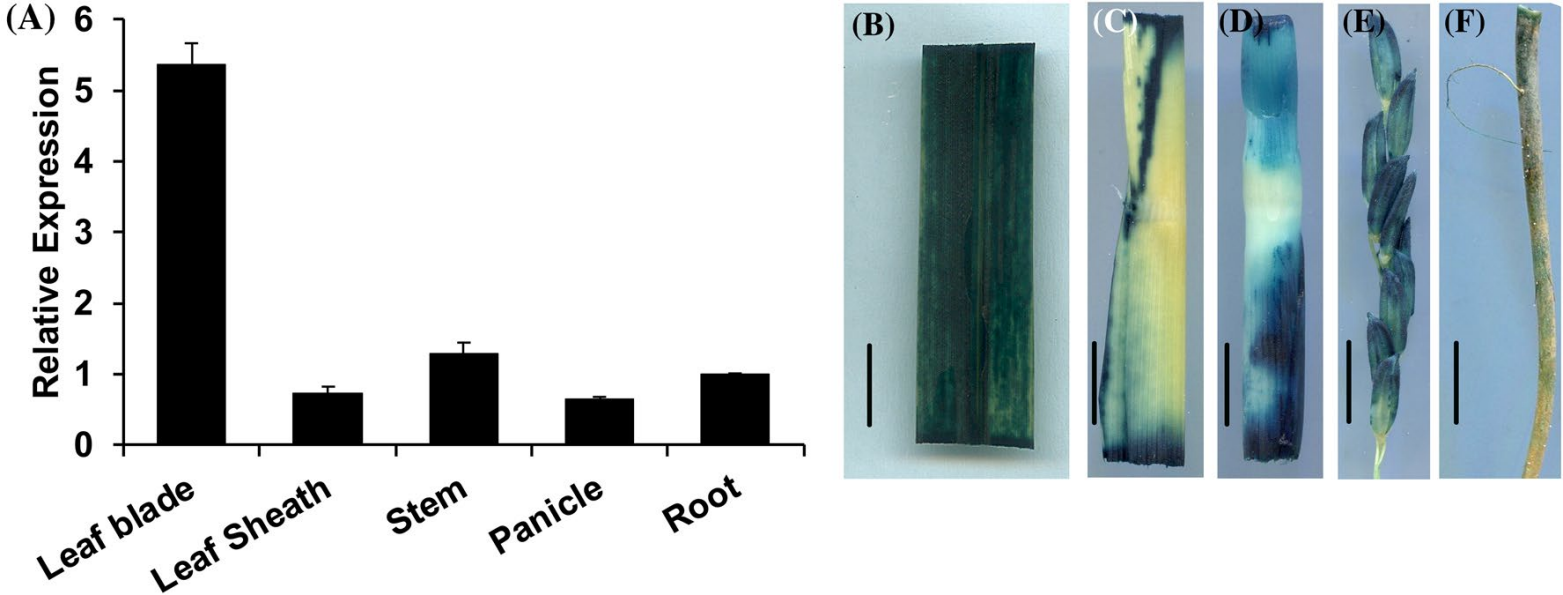
Expression analysis of *ES5*. **a** Transcriptional level of *ES5* at different organs- leaf blade, leaf sheath, stem, panicle and root. Values are mean ± SD of three biological replications. **b–f** GUS analysis of *ES5* expression in different parts of the plants. Scale bar = 1 cm

To determine the spatial and developmental expression pattern of *ES5* in rice qRT-PCR was conducted using specific primers. The mRNA levels of *ES5* were detected in different organs. As expected, *ES5* expressed constitutively across the tissue type but expressed preferentially in the leaf blade rather than leaf sheath, stem, panicle and root (Fig. 6a). In addition, to evaluate the spatial expression we expressed the GUS gene under the control of the native promoter of *ES5* gene. The *ES5*:GUS transgenic lines were analyzed and similar results were found. GUS activity was expressed in all tissues tested, which is consistent with the qPCR data (Fig. 6b–f). Moreover, *ES5* is expressed constituently throughout the whole life cycle of the plant (Fig. S6a, b).

### *ES5* encodes a phosphatidylserine synthase

The full length ES5 protein consists of 422 amino acids with an estimated molecular mass of 49.23 kDa. *ES5* encodes PSS which is an enzyme involved in serine exchange reaction (EC:2.7.8.8) (https://enzyme.expasy.org). The PSS protein family represents PSSI and PSSII, which are membrane-bound proteins involved in the catalytic replacement of the head group of a phospholipid (phosphatidylcholine or phosphatidylethanolamine) with l-serine (Kuge and Nishijima 1997). To deduce the evolutionary associations of ES5 homologs among other plant species a phylogenetic tree was built. The ES5 protein shares a high degree of resemblance with other plant species (Fig. S7).

Alignment of homologous protein among rice, Arabidopsis, maize and tobacco revealed that ES5 is highly conserved (Fig. S8a). ES5 contains six transmembrane domains, as predicted by TMHMM Server2.0, and the mutation occurred immediately after the start of the second transmembrane domain (Fig. S8b). According to SMART (https://smart.embl-heidelberg.de/) database ES5 contains two transmembrane domain and a PSS domain. There are other 4 transmembrane domains present which are overlapped with PSS domain. PSS domain starts from the amino acid position 99 and ends at position 380 (Fig. S8c). And the substitution and creation of premature stop codon occurs at the position 104. Thus, we inferred that the deletion of PSS domain of es5 should affect to its activity.

### Measurement of PSS and phospholipids

**Fig. 7:**
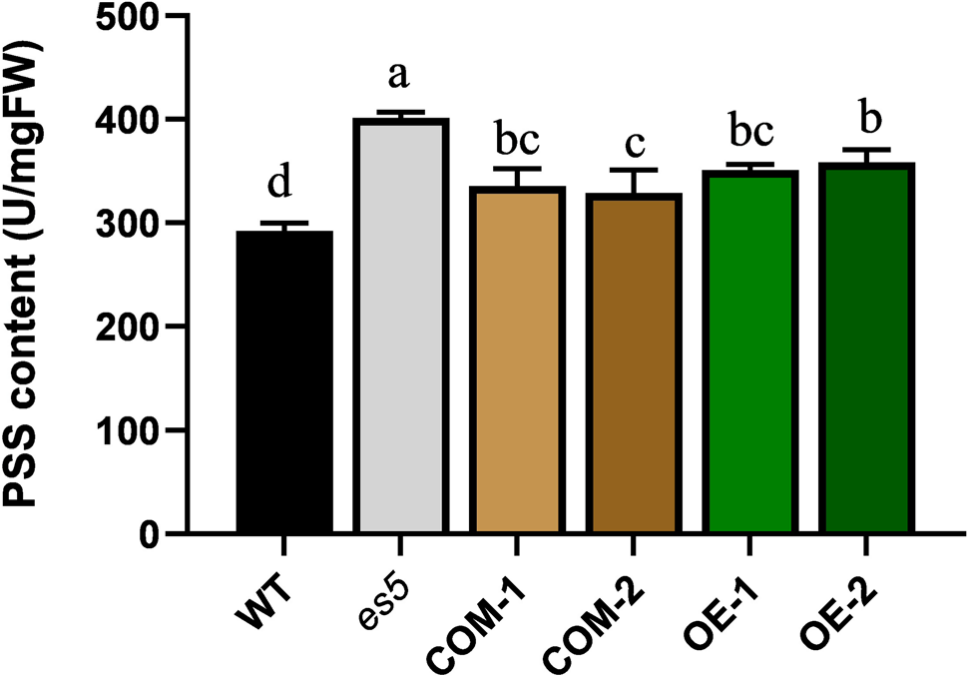
PSS content in WT, *es5*, complementation and overexpression lines in the flag leaves at flowering stage. Data = mean ± SD (n = 3). Letters in the figure indicate the result of multiple comparison test; a, b, c indicate the significant differences on 0.05 level

**Fig. 8:**
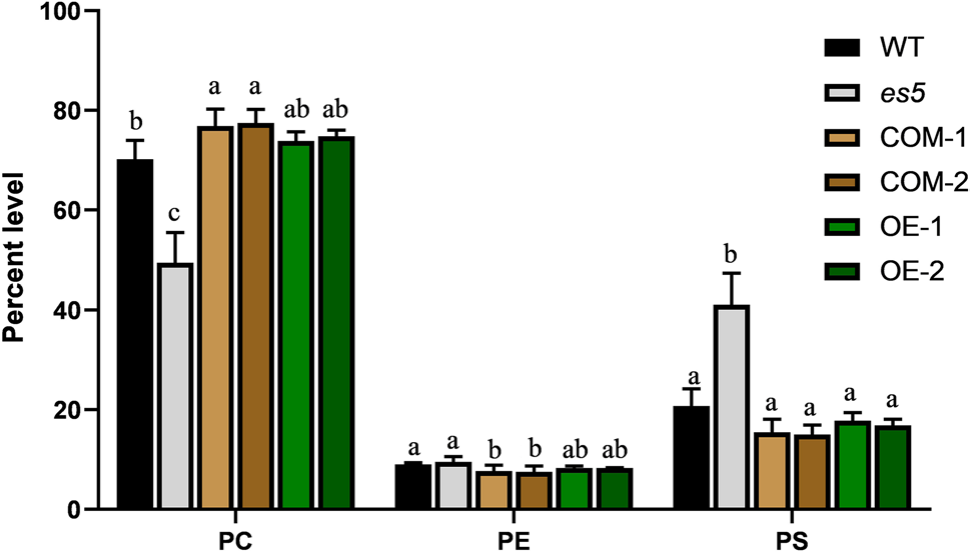
Phospholipids level of WT, *es5*, complementation and overexpression lines in the flag leaves at flowering stage. Data = mean ± SD (n = 3). Letters in the figure indicate the result of multiple comparison test; a, b, c indicate the significant differences on 0.05 level

The rice genome encodes three highly homologous phosphatidylserine synthase proteins (Fig. S8a). qRT-PCR revealed that the expression of the other two homologs viz. *SUI1* (LOC_Os01g02890) and *SUI3* (LOC_Os01g49024) are upregulated in *es5* (Fig. S9). PSS functions as a catalyzer to synthesize PS where PC or PE could be the possible substrate (Vance and Steenbergen 2005). We measured the total PSS content in the flag leaves of WT, *es5*, complemented and overexpression lines at the flowering stage. We found that total PSS content is higher in the *es5* mutant plants compared to wild-type, complementation and overexpression lines (Fig. 7). PSS content was 401.38 ± 5.63 U/mg FW in the *es5* plants whereas, it was 292.16 ± 7.7 U/mg FW in the wild-type plants which is significantly lower than *es5* plants. Among the complementation and overexpression lines PSS contents varied from 329.02 ± 22.22 and 358.42 ± 12.04 which were also significantly lower than the *es5* mutant plants (Table S2). We also measured the PC, PE and PS in the leaves of wild-type, *es5*, complementation and overexpression lines. PS level was significantly higher and PC level was significantly lower in the *es5* plants compared to the wild-type, complementation and overexpression lines (Fig. 8 and Table S3).

## Discussion

Senescence is the process or condition of getting old, ultimately ending in death. From the perspective of plant science, it is a positive regulator to support plant growth, differentiation, adaptation, survival and reproduction (Thomas 2013) which happens in a synchronized manner and under strict genetic control (Schippers 2015). Although it is a natural phenomenon that happens in a time-dependent manner, it can also be initiated early. Senescence tissue is viable and up until the onset of senescence the cell membrane and organelles retain their integrity. Senescence starts from the leaf tip and moves downwards (Thomas 2013). In a prematurely senescent plant, some important changes are observed throughout the plant life cycle: color change from green to yellow or brown, degradation of chloroplasts, reduction in chlorophyll contents and photosynthesis, degradation of proteins, nucleic acids and lipids, translocation of macro and micro molecules to the storage organs and reduction in yield-related attributes (Huang et al. 2016). In our study we have identified an early senescence mutant *es5* in which we have observed leaf color change from green to yellow, reduced chlorophyll content and photosynthesis, altered senescence-related parameters, altered expression of SAGs and lower performance of agronomic traits. Severe loss of photosynthetic activity and degradation of chlorophyll pigments in the *es5* plants at the maturity stage indicates the severity of the senescence phenotype. The chlorophyll- and photosynthesis-related genes also showed altered expression levels in mutant plants. Chlorophyll content and photosynthetic rate have a direct correlation with rice yield. As mentioned previously, rice yield is correlated with the number of effective tillers, thousand grain weight and seed setting percentage. In *es5* plants all these yield attributing traits were significantly reduced which caused poor yield compared to the wild-type. Moreover, mRNA levels of yield related genes like *GS3* was up-regulated and *GS5* was down-regulated in the *es5* mutant plants (Fig. S2).

Various metabolic processes produce ROS in plants, initiating oxidative damage to thylakoid membranes and other cellular components. The increase in the ROS such as H_2_O_2_ and O^2−^ is believed to be a cause of initiation of senescence. In the present study, DAB staining indicated that H_2_O_2_ is accumulated in *es5* plants. ROS is responsible for the peroxidation of membrane lipid MDA. In the *es5* plants there was an elevated level of MDA whose accumulation causes cellular membrane damage and indirectly affects cell death (Li et al. 2014). Cell death was confirmed by Evans blue staining. To survive under stressed conditions, plants eliminate the overproduction of ROS through scavenging enzymes like SOD and CAT (Tripathi et al. 2009). In *es5* plants, SOD production was higher compared to wild-type and this production was increased after 7 days of flowering. This indicates that the plants responded actively to the ROS production and transferred O^2−^ into H_2_O_2_ but the other scavenger CAT did not increase and as a consequences H_2_O_2_ accumulated in the *es5* mutant leaves and execution of senescence phenotype. Higher SOD and lower CAT level were also found in the *es3(t)* and *es4* mutants (Su et al. 2016; Wang et al. 2019).

Through mapping-based cloning we have proved that these changes occurred in the mutant due to one base substitution and creation of a premature stop codon of the *ES5* gene located on the long arm of chromosome 5. In rice, *ES5* was reported as *SUI2* which has two more homologous genes reported as *SUI1* (LOC_Os01g49024) and *SUI3* (LOC_Os01g02890) (Yin et al. 2013; Zhu et al. 2011). All these genes are reported for shortened uppermost internode phenotype. *ES5* (*SUI2*) is expressed in all plant tissues, preferentially in the leaf blade (Fig. 6) but the other two homologs are more heavily expressed in panicle, node and internode and rarely in leaves (Yin et al. 2013). In our *es5* mutant *SUI1* and *SUI3* are overexpressed, whereas *SUI2* is down-regulated (Fig. S9). Previously *SUI2* RNAi lines showed that *SUI2* has redundant function for both internode elongation and panicle expansion where other two homologs *SUI1* and *SUI3* were also down-regulated (Yin et al. 2013). Our *es5* mutant had reduced internode length (Fig. S10) accompanied with early senescence, implying that the SUI gene family may also control leaf senescence in rice. These SUI gene family encodes a membrane-bound protein PSS which is involved in the biosynthesis of phosphatidylserine. Although a minor phospholipid, PS plays an important role in many biological functions like cell growth, cell death signaling, vesicular trafficking, lipid-protein interaction and membrane lipid metabolism (Delhaize et al. 2002; Yamaoka et al. 2011). In plants, PS molecular species are produced in the ER and translocated to the plasma membrane (Moreau et al. 2002). PS might have an important role in normal growth and development and its reduction may cause reduced phenotype (Yin et al. 2013). On the other hand, PS accumulation leads to necrotic phenotypes (Delhaize et al. 2002). There are many species of PS which are categorized as long chain fatty acids (LCFA) and very long-chain fatty acids (VLCFA) (Li et al., 2014). Li et al. (2014) concluded from their experiments that VLCFA containing PS could be increased in plants due to their time-dependent accumulation during the lifespan of the plant and due to the response of cells to stress-induced damage. It implies that stressed condition and at the death of plants there is accumulation of VLCFA-PS. In the *es5* mutant total PS level is higher compared to wild-type, complementation and overexpression lines which may happen due to the up-regulation of *SUI1* and *SUI3*. Plants might have an internal mechanism to maintain the total PS levels at normal (Yin et al. 2013). Based on this hypothesis we speculate that may be *SUI1* and *SUI3* became active to guarantee PS homeostasis, but ultimately the PS content increased in the *es5* plants. Moreover, PC content was lower in *es5* plants and higher in the complementation and overexpression lines compared to the wild-type plants pointing that PC may be the product synthesized by *ES5* and there is also possibility that PS contributes to PC biosynthesis (Delhaize et al. 2002; Datko and Mudd 1988; Kinney and Moore 1987). PC is the main phospholipid in the outer membrane of the chloroplast (Botella et al. 2017). Decline in PC content is parallel to the aging of the plant and which may be an outcome of senescence (Itzhaki et al. 1998). Now, it is unclear whether PS or PC are correlated with cell death, and as abovementioned. It is also not clear how PS level is maintained at normal in plants and what is the functions of *ES5*, *SUI1* and *SUI3* in the PS homeostasis.

In summary, *es5* plants had increased levels of PS and decreased level of PC which may leads to an increase in the levels of ROS and MDA, mobilization of soluble proteins, upregulation of senescence and chlorophyll degradation-related genes and down-regulation of chlorophyll biosynthesis and photosynthesis-related genes, which eventually leads to premature senescence. These results indicate that PS may have a vital role in plant cell death signaling pathways and open a platform for further analysis of the relationship between phospholipid metabolism and leaf senescence. Further research is needed to explore why *ES5* homologs *SUI1* and *SUI3* are up-regulated in *es5* mutants and how these three genes function together for PS biosynthesis in plants.

## Electronic supplementary material

Below is the link to the electronic supplementary material.

Electronic supplementary material 1 (DOCX 1223 kb)

Electronic supplementary material 2 (DOCX 22 kb)
