# Supplementary material for: ES5 is involved in the regulation of phosphatidylserine synthesis and impacts on early senescence in rice (Oryza sativa L.)
Source: Plant Mol Biol. 2020 Jan 9;102(4):501–15. doi: 10.1007/s11103-019-00961-4 (PMC7026238; doi:10.1007/s11103-019-00961-4)
Supplement: Supplementary file 1 — Electronic supplementary material 1 (DOCX 1223 kb) [file 11103_2019_961_MOESM1_ESM.docx]

*ES5* is involved in the regulation of phosphatidylserine synthesis and impacts on early senescence in rice (*Oryza sativa* L.)

Mohammad Hasanuzzaman Rani^1,2,3#^. Qunen Liu^1,2#^. Ning Yu^1,2^. Yingxin Zhang^1,2^. Beifang Wang^1,2^. Yongrun Cao^1,2^. Yue Zhang^1,2^. Md Anowerul Islam^1,2^. Workie Anley Zegeye^1,2,4^. Liyong Cao^1,2*^. Shihua Cheng^1,2*^

^1^ State Key Laboratory of Rice Biology, China National Rice Research Institute, Hangzhou, Zhejiang, 310006, China

^2^ China National Center for Rice Improvement, China National Rice Research Institute, Hangzhou, Zhejiang, 310006, China

^3^ Bangladesh Institute of Nuclear Agriculture, Mymensingh-2202, Bangladesh

^4^ Department of Plant Sciences, University of Gondar, Ethiopia

^#^Mohammad Hasanuzzaman Rani and Qunen Liu equally contributed to this work.

Corresponding authors

* Shihua Cheng, Tel: 86-571-63370188, [chengshihua@caas.cn](mailto:chengshihua@caas.cn)

* Liyong Cao, Tel: 86-571-63370329, [caoliyong1966@163.com](mailto:caoliyong1966@163.com)


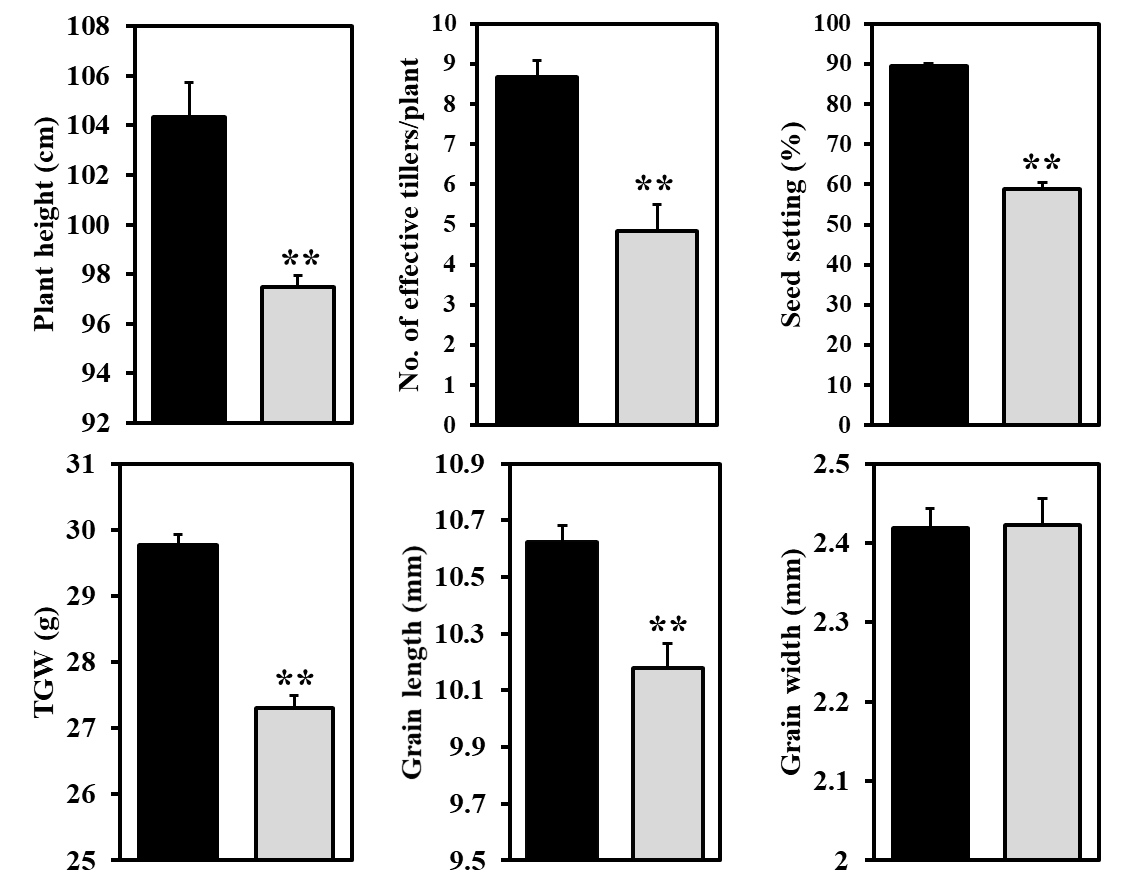


**Fig S1** Comparisons of agronomic traits between wild-type and *es5* plants. Black and grey bar represents wild-type and *es5* plants respectively. Values are mean ± SD of three biological replicates; p ≤ 0.01; Student’s *t*-test.

**Fig S2** Relative expression of grain size related genes between wild-type and *es5* plants. Black and grey bar represents wild-type and *es5* plants respectively. Values are mean ± SD of three biological replicates; p ≤ 0.01; p ≤ 0.05; Student’s *t*-test.


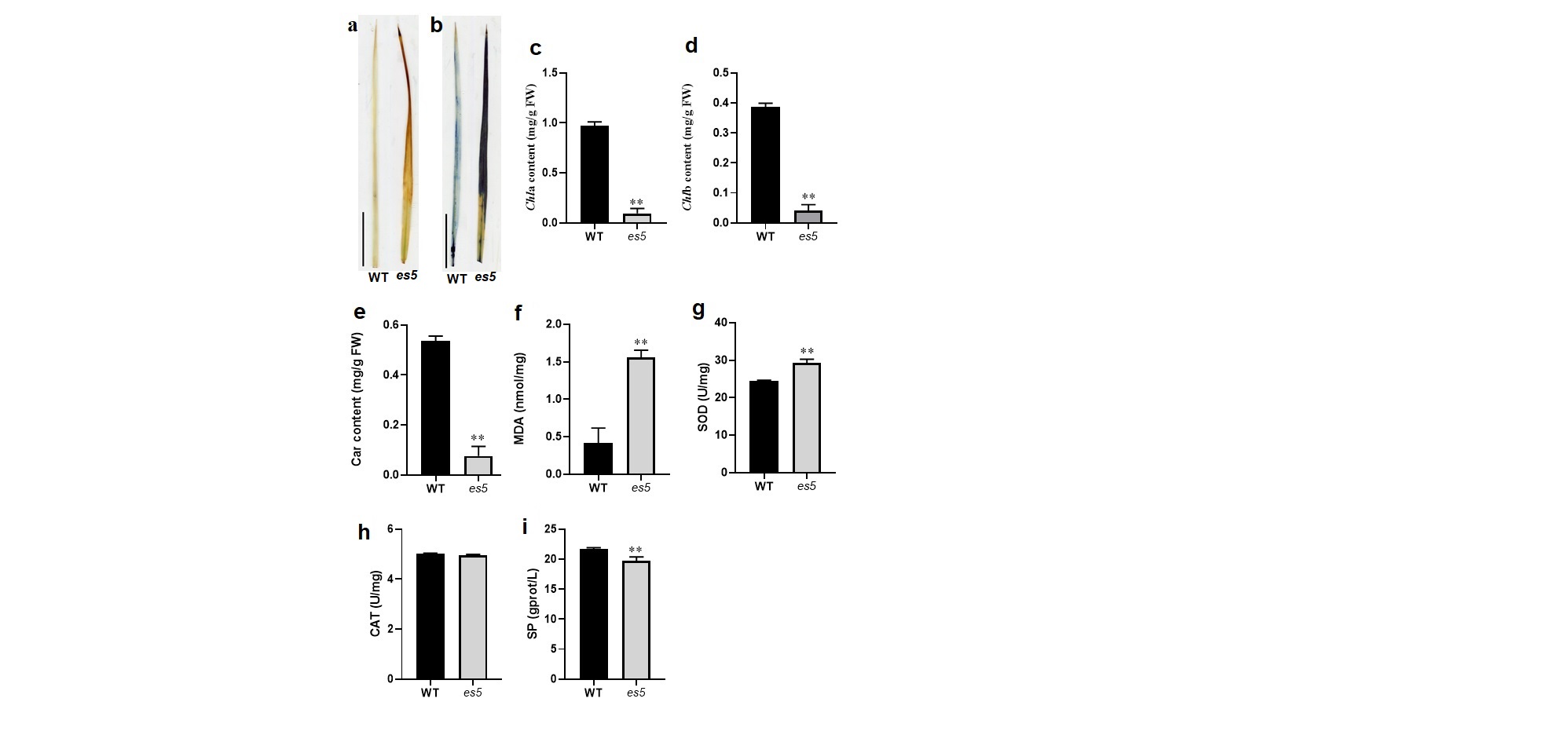


Fig S3 Histochemical and physiological characterization of wild-type and *es5* mutant leaves at the 4-leaf stage. a. DAB staining; b. Evans blue staining; bar = 2cm; c, d, e, f, g, h & i denotes. Chl*a*, Chl*b*, Carotenoid; MDA, SOD, CAT and SP content, respectively. Values are mean ± SD of three biological replicates; p ≤ 0.01; Student’s *t*-test.


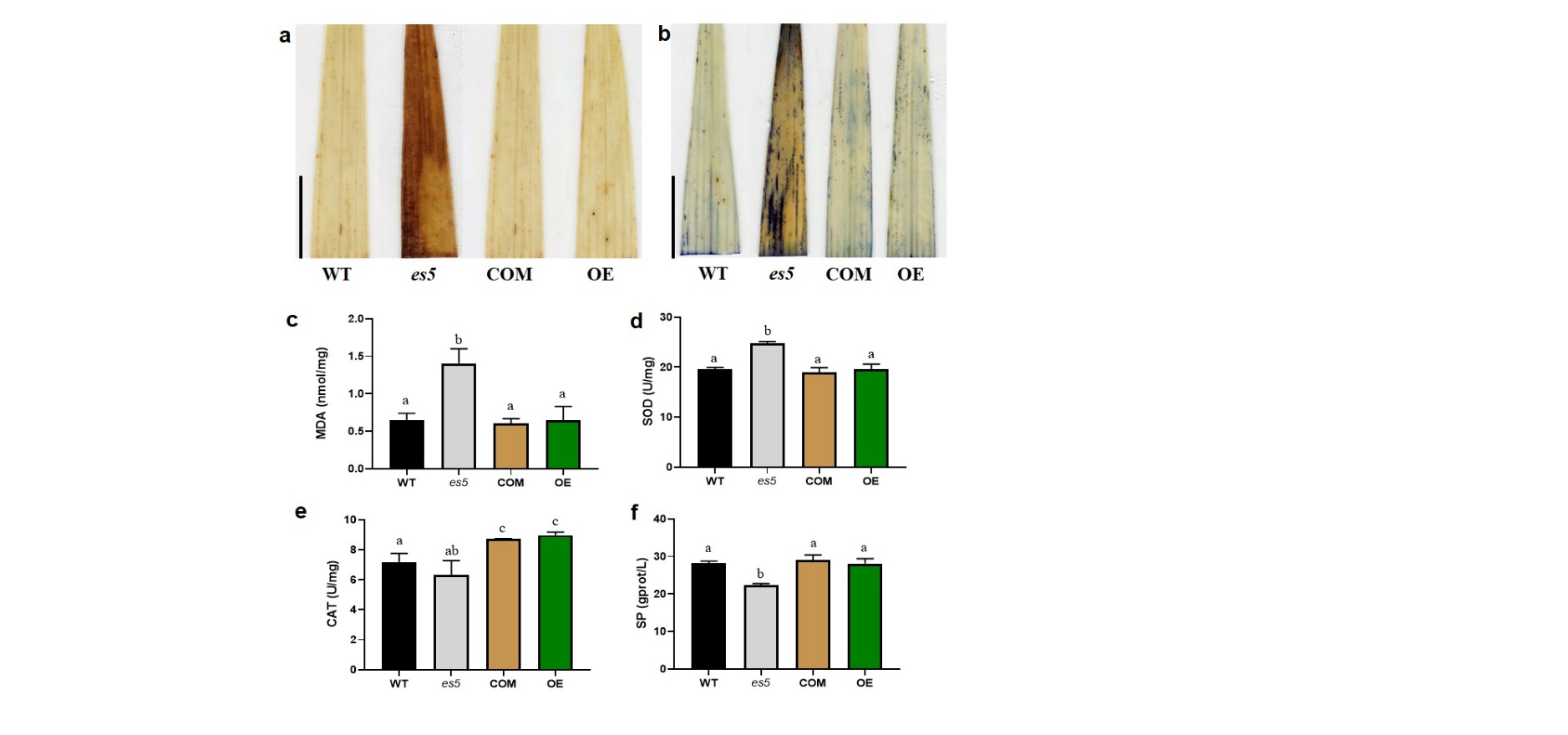


Fig S4 Histochemical and physiological characterization of wild-type, es5 mutant, complemented and overexpression leaves. a. DAB staining; b. Evans blue staining; bar = 2cm; c, d, e & f denotes MDA, SOD, CAT and SP content, respectively. Data=mean ± SD (n=3). Letters in the figure indicate the result of multiple comparison test; a, b, c indicate the significant differences on 0.05 level.


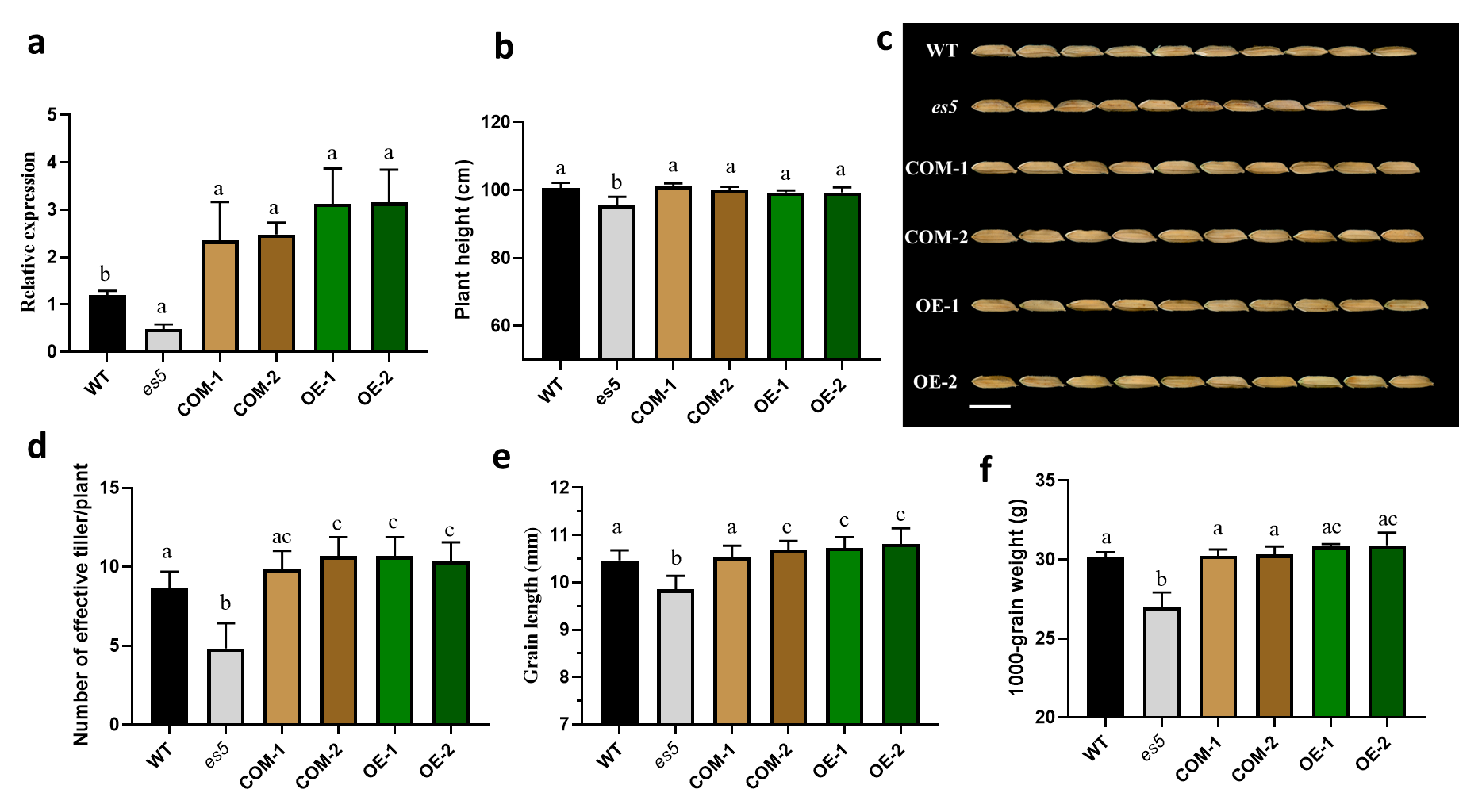


Fig S5 Comparison of agronomic traits of wild-type, *es5*, complemented and overexpression plants. a. relative expression of *ES5*; b, d, e & f denotes plant height, number of effective tiller/plants, grain length and 1000-grain weight, respectively; c. grain of different genotypes; bar = 10 mm. Data=mean ± SD (n=3). Letters in the figure indicate the result of multiple comparison test; a, b, c indicate the significant differences on 0.05 level.


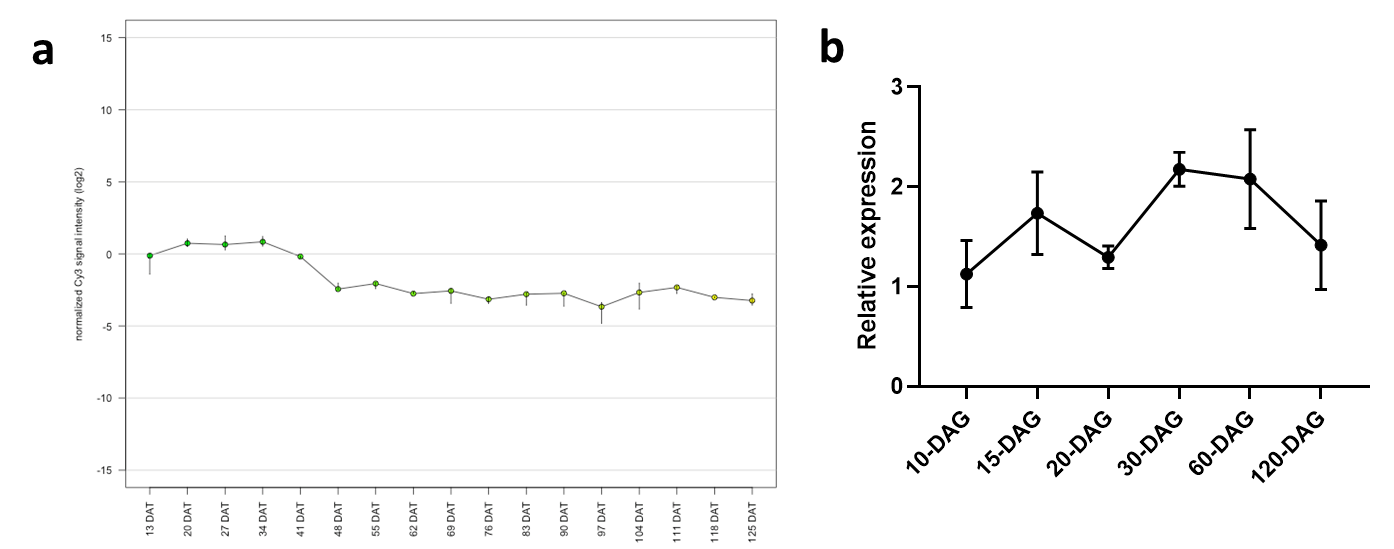


Fig S6 Expression pattern of *ES5* at different stage. a. Expression pattern obtained from public database RiceXPro; b. relative expression at different developmental stage; Values are mean ± SD of three biological replicates.


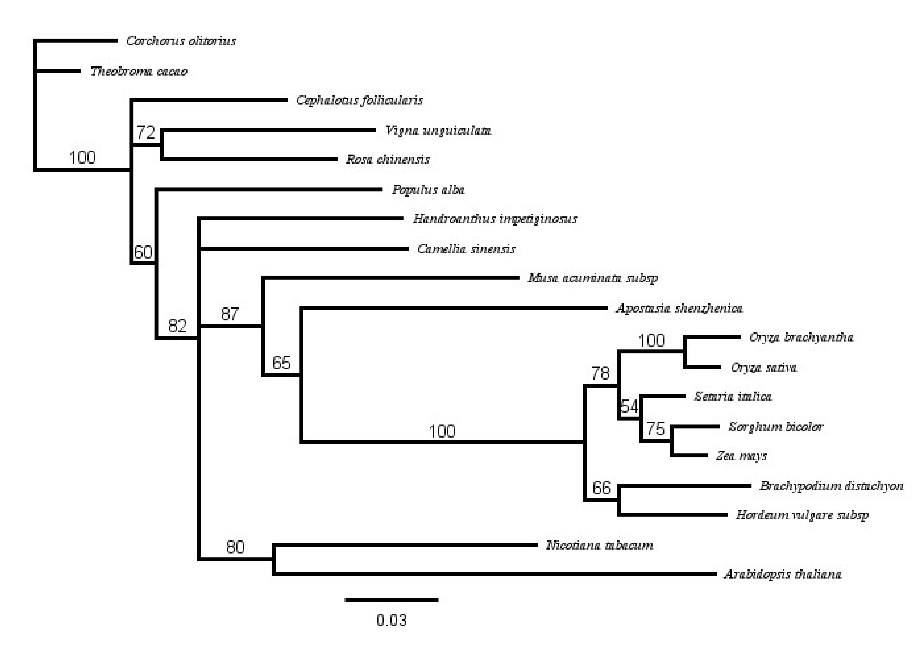


**Fig S7** Phylogenetic tree of ES5-like protein among different species. The numbers at each node represent the bootstrap support (percentage), and scale bar is an indicator of genetic distance based on branch length.


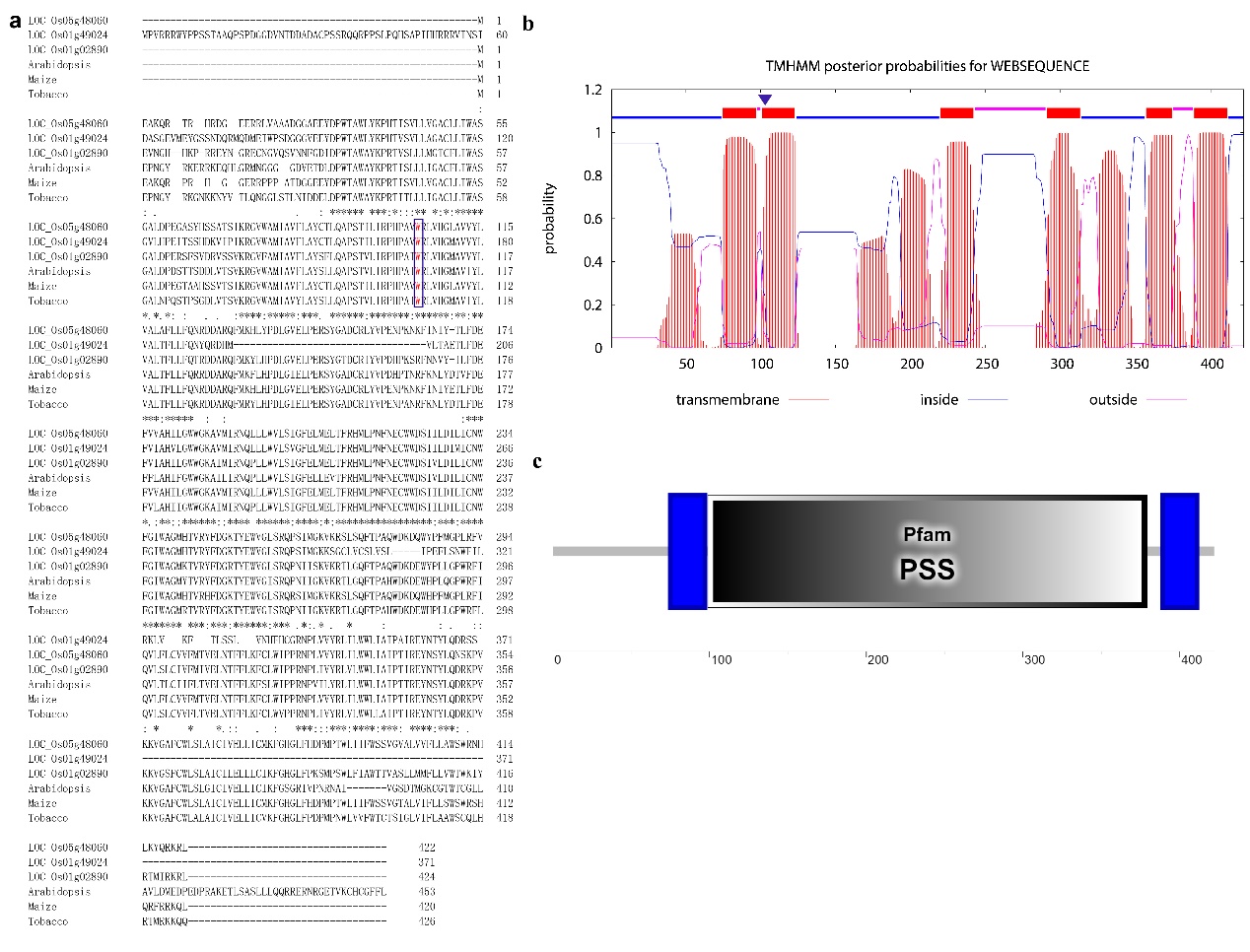


**Fig S8** (a). Multiple sequence alignments of the deduced amino-acid sequence of ES5 and its homologs. The mutation site in the amino acid sequence (highlighted) is at the position of 104 (sequence alignment done by ClustalO software). Residues that are conserved across all sequences are highlighted. Below the protein sequences is a key denoting conserved sequence (*), conservative mutations (:), semi-conservative mutations (.), and non-conservative mutations (). (b) TMHMM v2.0 topology prediction for ES5. Six transmembrane domains were predicted. (c) Domains of ES5 protein derived from SMART database.

**Fig S9** Relative expression of *ES5* (*SUI2*) and its homologs in wild-type, *es5*, complementation and overexpression lines. Values are mean ± SD of three biological replicates.


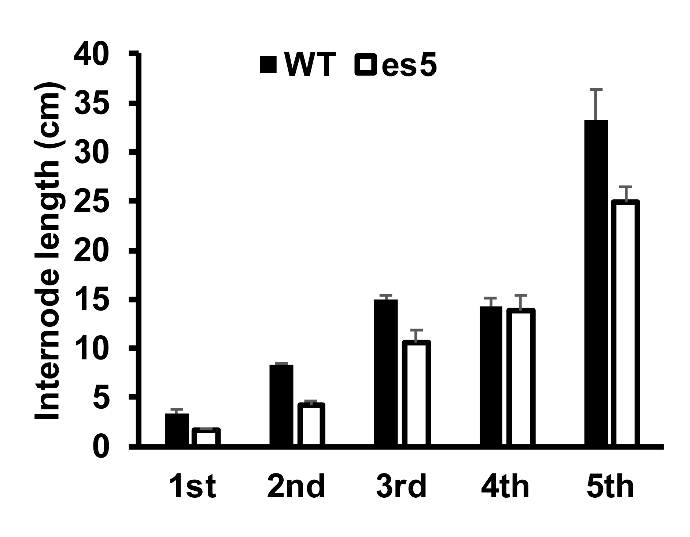


**Fig S10** Comparison of internode length between wild-type and *es5* plants. Values are mean ± SD of three biological replicates.
