# Supplementary material for: ES5 is involved in the regulation of phosphatidylserine synthesis and impacts on early senescence in rice (Oryza sativa L.)
Source: Plant Mol Biol. 2020 Jan 9;102(4):501–15. doi: 10.1007/s11103-019-00961-4 (PMC7026238; doi:10.1007/s11103-019-00961-4)
Supplement: Supplementary file 2 — Electronic supplementary material 2 (DOCX 22 kb) [file 11103_2019_961_MOESM2_ESM.docx]

*ES5* is involved in the regulation of phosphatidylserine synthesis and impacts on early senescence in rice (*Oryza sativa* L.)

Mohammad Hasanuzzaman Rani^1,2,3#^. Qunen Liu^1,2#^. Ning Yu^1,2^. Yingxin Zhang^1,2^. Beifang Wang^1,2^. Yongrun Cao^1,2^. Yue Zhang^1,2^. Md Anowerul Islam^1,2^. Workie Anley Zegeye^1,2,4^. Liyong Cao^1,2*^. Shihua Cheng^1,2*^

^1^ State Key Laboratory of Rice Biology, China National Rice Research Institute, Hangzhou, Zhejiang, 310006, China

^2^ China National Center for Rice Improvement, China National Rice Research Institute, Hangzhou, Zhejiang, 310006, China

^3^ Bangladesh Institute of Nuclear Agriculture, Mymensingh-2202, Bangladesh

^4^ Department of Plant Sciences, University of Gondar, Ethiopia

^#^Mohammad Hasanuzzaman Rani and Qunen Liu equally contributed to this work.

Corresponding authors

* Shihua Cheng, Tel: 86-571-63370188, [chengshihua@caas.cn](mailto:chengshihua@caas.cn)

* Liyong Cao, Tel: 86-571-63370329, [caoliyong1966@163.com](mailto:caoliyong1966@163.com)

**Table S1.** List of primers used in the experiment

| **Primer name** | **Sequence** |
| --- | --- |
| Fine mapping | |
| BF-20F | CCACTGAATTCCAGCTCTTTGACC |
| BF-20R | GCCTGGTGTTCTTGCTGTTGC |
| 2H-31F | ATCGAGCCCAACCCAATT |
| 2H-31R | CAAGAGCGACAGGCAACA |
| 2H-3F | AAAGCTCCGTCGGCCTGAT |
| 2H-3R | TCGCCTTCGCTTACTCACA |
| 4H-6F | GCTTTTGGCTCATGGCTAG |
| 4H-6R | CTGATCCCTGTAGGGCTGT |
| 3H-2F | AATATGAGTTGATTTGCTTCAAT |
| 3H-2R | TGCCTGATGAGTTCTTTGCT |
| 3H-6F | CATAGCTCAGCTTTTCTTCTTT |
| 3H-6R | ATCCAAATGCTACAGATGACG |
| 3H-12F | CCGAGTGAAGGGAAATGGA |
| 3H-12R | AGCCTCAAAGCACCAAACA |
| 2H-15F | TAGATGATTGGTAGCAGGAAAA |
| 2H-15R | ACGTAAAAGTAAAGTAAGGGAAA |
| 2H-28F | GCGAAATCAGAAGGTAGCC |
| 2H-28R | GACCAGTCCTTTCACATTGGT |
|  |  |
| Primer used for vector construction | |
| COM-F | CCATGATTACGAATTCATCGTATATGTGCATCTTTCGT |
| COM-R | GGCCAGTGCCAAGCTTTAAACTTTTCATCGAGCACCT |
| OE-F | CGGTCCCGGGGGATCCATGGAGGCGAAGCAGAGGACG |
| OE-R | TGCTCACCATGGATCCTAGCCGCTTTCTCTGATATTTCAGG |
| GUS-F | CGGTACCCGGGGATCCGCGTGGAAAACAGTCGCTTT |
| GUS-R | CTCAGATCTACCATGGGTTCCCACTTCCAGAACCCTC |
|  |  |
| Primers used for qPCR | |
| Osh36-F | GCACGGAGGCGAACGA |
| Osh36-R | TTGAGCGGTAGCACCCATT |
| OsI85-F | GAGCAACGGCGTGGAGA |
| OsI85-R | GCGGCGGTAGAGGAGATG |
| rbcL-F | CTTGGCAGCATTCCGAGTAA |
| rbcL-R | ACAACGGGCTCGATGTGATA |
| SGR-F | AGGGGTGGTACAACAAGCTG |
| SGR-R | GCTCCTTGCGGAAGATGTAG |
| Osl57-F | ACCCTAAAGTAAATGAAGTC |
| Osl57-R | CCTGCTCTTGTCTTGTTA |
| Cab1R-F | AGACGTTCGCCAAGAACC |
| Cab1R- R | GAGGAGCTCCGGGAAGAC |
| CHLD-F | GGAAAGAGAGGGCATTAG |
| CHLD-R | CAATACGATCAAGTAAGTGTT |
| RCCR1-F | CGCATTTCCTCATGGAATTT |
| RCCR1-R | CTTCTCACGCTGTTTGTCCA |
| SUI1-F | CTGATCATCCGAAAAGCAGGTT |
| SUI1-R | AAGGATATGGGCAATAACAAACTCAT |
| ES5-F | TGGGCAAGGTGAAAAGATCAC |
| ES5-R | GCAACGGCCCCATGAAA |
| SUI3-F | GGGTCCTTCATCCAGAGATTAC |
| SUI3-R | TGCTTGGAGAGTGCAGTATG |
| ActinF | CAGGCCGTCCTCTCTCTGTA |
| ActinR | AAGGATAGCATGGGGGAGAG |

**Table S2** Total PSS contents

| WT | *es5* | COME-1 | COM-2 | OE-1 | OE-2 |
| --- | --- | --- | --- | --- | --- |
| 292.16 | 401.38 | 335.76 | 329.02 | 351.16 | 358.42 |
| 7.70 | 5.63 | 16.44 | 22.22 | 5.31 | 12.04 |

**Table S3** Phospholipid contents

| Plant materials | Phospholipids (%) | | |
| --- | --- | --- | --- |
|  | PC | PE | PS |
| WT | 70.22 ± 3.75 | 9.05 ± 0.31 | 20.73 ± 3.47 |
| *es5* | 49.41 ± 6.08 | 9.5 ± 1.10 | 41.09 ± 6.21 |
| COM-1 | 76.84 ± 3.42 | 7.71 ± 1.12 | 15.46 ± 2.60 |
| COM-2 | 77.45 ± 2.77 | 7.52 ± 1.15 | 15.03 ± 1.87 |
| OE-1 | 73.87 ± 1.82 | 8.32 ± 0.35 | 17.81 ± 1.63 |
| OE-2 | 74.82 ± 1.19 | 8.3 ± 0.043 | 16.88 ± 1.22 |
